# Preliminary Announcement and Program State Medical Association

**Published:** 1895-04

**Authors:** 


### Society Notes.

### TEXAS STATE MEDICAL ASSOCIATION.

### Preliminary Announcement and Programme of the Twenty-Seventh Annual Meeting.

ANNOUNCEMENT.

The "Texas State Medical Association" will hold its twenty-seventh annual session in Dallas, Texas, April 23d to 26th, inclusive. All reputable physicians are respectfully and urgently invited to attend, and aid the Association in holding up the standard of scientific medicine in this State.

Reports from all portions of the State encourage the hope that this meeting will mark an epoch in the history of the Association, and that there will be made an earnest and, we trust, a successful effort, to unite the separate medical organizations of Texas as members of a grand State Medical Association.

The medical profession of the State has just cause to be proud of its local medical societies, and of the excellent work they have done. They should be encouraged and fostered, and should carry the membership of every eligible physician within their territory; but they should not be regarded as rivals of the State Association.

Many valuable papers have been promised this meeting by well known physicians of this State, and distinguished medical men from other States have accepted our invitation to be present and read papers. Altogether, there are good reasons for believing that the meeting at Dallas will not disappoint those who look forward to the occasion as a "feast of reason," nor are assurances lacking that our hospitable friends in Dallas will make it a "flow of soul":—There will be at least food for thought, and rest and entertainment for the body.

Never before has scientific medicine received an equal amount of encouragement and intelligent recognition by the thinkers of the world, as it has within the past decade. These have been called forth by the exact and scientific methods that have been pursued in the investigations of medical questions,—and to the brilliant achievements of medicine within this period. It is safe to say that the value of medical discoveries within this time, if measured by the amount of good they will do for man, are not excelled by those in any other branch of science.

The records will bear me out in saying that Texas physicians have been no laggards in this race, but the record would be far more valuable if it included the unrecorded work that has been done by Texas physicians who claim to be too busy to write. We have an abundance of recorded clinical hospital observation, but an insufficiency from the rural districts. The meeting at Dallas will give the busy country doctor an opportunity to compare his medical work and results with those of his city brothers, and it is hoped the opportunity will not be neglected.

In conclusion, permit me, doctor, to urge you to attend the Dallas meeting and assist in making "The Texas State Medical Association" the representative Association of the medical profession of the entire State. We should remember that "In Union there is Strength."

J. W. McLaughlin, M. D.,
President T. S. M. A.

---

The Committee of Arrangements reports as follows:

Transportation.—Information has been received from Mr. J. S. Keenan, General Passenger Agent, of the G., C. & S. F. R. R., who kindly took the matter in charge, that the railroads of the State have agreed to make a rate of one and one-third fare for the round trip upon the certificate plan. Passengers paying full fare, going to the meeting, and securing a receipt for the same. In order to be good for return trip at one-third fare, this receipt must be signed by Dr. H. A. West, Secretary, and stamped by Mr. W. G. Wilkens, Passenger Agent G., C. & S. F. R. R., at Dallas, who has been made joint agent to represent the other roads. Delegates are requested to present their certificates upon the first day of the meeting.

Hotel Rates.—The hotels of Dallas have agreed to make a reduction in rates, but as details have not been received, they will have to be published later.

### PRELIMINARY PROGRAMME.

The session will begin Tuesday morning, April 23d, at 11 o'clock. The place of meeting will be the City Hall. Members are requested to assemble early, in order to register, as far as possible, before the opening. The Secretary, Treasurer, and Reception Committee, will be present to attend to registration. Applicants for membership will sign blank forms, giving postoffice address and county. Each application must have the signatures of two members, and be accompanied by the initiation fee, $5, and dues for one year, $5, also by the diploma or other satisfactory evidence of graduation from a regular medical college. Applicants will receive badges when they exhibit the Treasurer's receipt. Members will receive badges after registration, and they are urgently requested to register promptly. The badge is an evidence of membership, and will not be given others.

The meeting will be called to order by the President, and prayer will be offered by Rev. C. T. Scofield.

Dr. McReynolds, on behalf of the local profession, will deliver the address of welcome.

As the titles of papers appearing in this programme were received by the Secretary in time to be arranged in their proper places, they will take precedence of those offered subsequently. Several titles of papers, prepared and to be offered, have been received from gentlemen who are not members of this Association, nor of affiliating societies, and not accredited by members of either. Such titles will receive recognition after application of the authors for membership have been favorably acted upon by the Judicial Council.

### SECTION ON PRACTICE OF MEDICINE.

1. Report of Chairman J. W. Carhart, M. D., La Grange.
2. The value to the general practitioner of a knowledge of nervous and mental disease.—Landon Carter Gray, M. D., New York.
3. The treatment of dysentery.—H. A. West, M. D., Galveston.
4. The climatic causation of disease, and the distribution of certain diseases in Texas.—I. M. Cline, M. D., Galveston.
5. Enteric fever.—W. B. McKnight, M. D., Springtown. Discussion opened by H. A. West. M. D., Galveston, and J. W. Burch, M. D., Aurora.
6. The healing power of drugs.—Q. C. Smith, M. D., Austin.

7. Phenacitin as a toxic agent.—David Cerna, Ph. D., M. D., Galveston.

8. The importance of post nasal catarrh as applied to general practice.—Wm. Caston, M. D., Corsicana.

9. Asthma, its causation and treatment, with report of cases. —J. W. Hunter, M. D., Waco. Discussion opened by Dr. L. Ashton, Dallas.

J. H. Frey, M. D., Sec'y, Corsicana.

SECTION ON OBSTETRICS AND DISEASES OF CHILDREN.

1. Report of Chairman A. B. Gardner, M. D., Bellville.

2. Report of two cases of tubal pregnancy.—J. E. Thompson, M. D., Galveston.

3. Maternal impressions.—G. W. Parker, M. D., Houston. Discussion opened by J. E. Gibson, M. D., McKinney.

A. H. Schenck, M. D., Sec'y, Kinney, Texas.

SECTION ON SURGERY.

1. Report of Chairman A. C. Walker, M. D., Fort Worth.

2. One month's work at the Randall Island Hospital, with remarks on ether anæsthesia in children.—Sam'l E. Milliken, M. D., New York.

3. Establishment of a permanent artificial opening for the relief of impermeable organic stricture of the urethra.—St. Cloud Cooper, M. D., Jefferson.

4. Tetanus.—P. C. Coleman, M. D., Colorado.

5. Laparotomy: Report of a case.—E. A. Jones, M. D., Osage.

6. Prostatic troubles and their treatment.—J. P. Oliver, M. D., Caldwell.

7. Fecal fistulæ and artificial anus.—J. E. Thompson, M. D., Galveston.

8. Paper, title not given.—C. A. Smith, M. D., Tyler.

E. D. Capps, M. D., Sec'y, Fort Worth.

SECTION ON MEDICAL JURISPRUDENCE.

1. Report of Chairman F. S. White, M. D., Houston.

2. Theoretical reflection upon increase of mental unsoundness in Christendom.—D. R. Wallace, M. D., Waco.

B. F. Church, M. D. Sec'y, Terrell.

SECTION ON STATE MEDICINE, ETC.

1. Report of Chairman R. Rutherford, M. D., Houston.

2. Ophthalmia neonatorum.—J. O. McReynolds, Dallas. Discussion opened by Dr. J. W. Carhart, La Grange.

3. Specialism in medicine.—Wm. Caston, M. D., Corsicana.

I. N. SUTTLE, M. D., Sec'y, Corsicana.

SECTION ON DERMATOLOGY.

1. Report of Chairman S. E. Hudson, M. D., Austin.

2. Eczema.—F. E. Daniel, M. D., Austin.

M. M. SMITH, M. D., Sec'y, Austin.

SECTION ON GYNECOLOGY.

1. Report of Chairman J. E. Gilchrist, M. D., Gainesville.

2. Etiology of cystic tumors of the uterus and adnexa.—Wm. H. Wathen, M. D., Louisville, Ky.

3. Therapeutic value of certain tonics in cancers of the uterus. —G. Wiley Broome, M. D., St. Louis. Mo.

4. Hæmatocele: Report of a case.—S. P. Kirkpatrick, M. D., Commerce, Texas. Discussion opened by J. J. Dial. M. D., Sulphur Springs.

5. Dysmenorrhœa.—John Shade Turner, M. D., Granbury. Discussion opened by J. H. Florence, M. D., Dallas.

6. Report of four laparotomies, including one case of hysterectomy and one of appendicitis.—J. Cummings, M. D., Austin.

7. Fibroid tumor of the womb: Report of a case.—J. W. Carhart, M. D., La Grange.

8. Surgical treatment of uterine fibroids.—R. E. Haughton, Midland, Texas.

9. Modern method of educating girls.—J. T. Wilson, M. D., Sherman.

10. Currettage in pelvic inflammations.—J. F. Y. Paine, M. D., Galveston.

E. L. MENEFEE, M. D., Sec'y, Granbury.

SECTION ON OPHTHALMOLOGY, ETC.

1. Report of Chairman R. E. Moss, M. D., San Antonio.

2. Nasal reflexes.—By Edward J. Bermingham, A. M., M. D., surgeon-in-chief to the New York Throat and Nose Hospital, New York.

3. The relative importance of various affections in the promotion of impaired vision.—J. O. McReynolds, M. D., Dallas.

4. Syphilitic ophthalmia—H. L. Hilgartner, M. D., Austin.

5. The care and preservation of the eyes.—Frank W. Boyd, M. D., San Antonio.

6. Otitis media.—W. T. Cole, M. D., Waco.

7. Otitis media: Report of cases.—R. H. Clinton, M. D., Dallas.

Ophthalmia neonatorum and some suggestions in regard to legislation.—W. P. Davis, M. D., Houston.

SECTION ON MICROSCOPY AND PATHOLOGY.

1. Report of Chairman Allen J. Smith, M. D., Review of recent work in pathology.

2. Further remarks upon parasites of man encountered in Texas.—F. Herff, M. D., San Antonio.

3. The antitoxin of diphtheria in practice. Reports of several cases.—Wm. Gammon, M. D., Galveston.

H. A. WEST, M. D., General Secretary,
2020 Market St., Galveston.

